# Severe immunodeficiency spectrum associated with *NHEJ1* gene mutation: Cernunnos/XLF deficiency

**DOI:** 10.7705/biomedica.7414

**Published:** 2024-12-23

**Authors:** Ana María Navarro, Gabriela Mantilla, Jorge Andrés Fernández, Mario Fernando Unigarro, Alfonso Suárez, María Claudia Ortega

**Affiliations:** 1 Hospital Infantil Universitario de San José, Fundación Universitaria Ciencias de la Salud - FUCS, Bogotá, D. C., Colombia Hospital Infantil Universitario de San José Hospital Infantil Universitario de San José Bogotá, D. C. Colombia; 2 Servicio de Genética Médica, Hospital Infantil Universitario de San José, Fundación Universitaria Ciencias de la Salud - FUCS, Bogotá, D. C., Colombia Hospital Infantil Universitario de San José Hospital Infantil Universitario de San José Bogotá, D. C. Colombia; 3 Servicio de Inmunología y Alergología, Fundación Universitaria Ciencias de la Salud - FUCS, Bogotá, D. C., Colombia Fundación Universitaria Ciencias de la Salud - FUCS Fundación Universitaria Ciencias de la Salud - FUCS Bogotá, D. C. Colombia

**Keywords:** Severe combined immunodeficiency, DNA end-joining repair, immunogenetics, genetics, heredity, syndrome, inmunodeficiencia combinada grave, reparación del ADN por unión de extremos, inmunogenética, genética, herencia, síndrome

## Abstract

Cernunnos/XLF deficiency is a rare, severe combined immunodeficiency, inherited in an autosomal recessive pattern (OMIM number: 611290), related to the *NHEJ1* gene. This gene participates in the DNA non-homologous end-joining pathway, repairing double-strand breaks in the DNA of mammalian cells. The clinical features include growth retardation, microcephaly, triangle-shaped face, recurrent infections, fibroblast’s excessive sensitivity to gamma-ionizing radiation, and hypogammaglobulinemia; also, low counts of subpopulations of B and T lymphocytes, with normal values of natural-killer cells.

This manuscript aims to present an extremely rare case of combined immunodeficiency in a twenty-years-old man with non-consanguineous parents and a homozygote variant of the *NHEJ1* gene. This case is the fiftieth reported in the literature and the first in Colombia, given the low prevalence of *NHEJ1*-related immunodeficiency and its difficult diagnosis due to scarce knowledge.

## Resumen

La deficiencia de Cernunnos XLF es una inmunodeficiencia combinada grave y poco frecuente, heredada de forma autosómica recesiva (número OMIM: 611290), relacionada con el gen *NHEJ*. Este gen participa en la vía de unión de extremos no homólogos reparando rupturas del ADN de doble cadena en las células de mamíferos. Las características clínicas de la deficiencia de Cernunnos XLF incluyen retraso del crecimiento, microcefalia, cara en forma de triángulo, infecciones recurrentes, sensibilidad excesiva de los fibroblastos a la radiación ionizante gamma, hipogammaglobulinemia y recuentos bajos de subpoblaciones de linfocitos B y T, pero valores normales de células *natural killer*.

El objetivo de este manuscrito es presentar un caso extremadamente raro de inmunodeficiencia combinada en un hombre de veinte años, hijo de padres no consanguíneos, que tiene una variante homocigota del gen *NHEJ1*. Este es el caso número 50 reportado en la literatura y el primero en Colombia, dada la baja prevalencia de la inmunodeficiencia y las dificultades en su diagnóstico por desconocimiento de la enfermedad.

Severe combined immunodeficiencies are a group of disorders affecting humoral and cell-mediated immunity ^1^. The International Union of Immunological Societies classifies Cernunnos/XLF deficiency as a primary severe combined T^-^/B^-^/NK^+^ immunodeficiency ^2^ - inherited in an autosomal recessive pattern- related to the *NHEJ1* gene mutation (OMIM number: 611290). This gene participates in the DNA non-homologous end-joining pathway. It repairs double-strand DNA breaks in mammalian cells and corrects programming errors during the V(D)J recombination of T and B lymphocyte antigen receptors for immunologic development ^3^.

The clinical features of Cernunnos/XLF deficiency include growth retardation, microcephaly, dysmorphic features, triangle-shaped face, recurrent infections, fibroblast’s excessive sensitivity to gamma ionizing radiation, hypogammaglobulinemia, and low counts of B and T lymphocytes, with normal values of natural killer (NK) cells ^4^^-^^6^. This disease has a very low prevalence: 0.1 per 100,000 born alive, with 49 cases reported in the literature ^4^.

This manuscript aimed to present an extremely rare case of combined immunodeficiency. This case is the fiftieth reported in the literature and the first in Colombia, given the low prevalence of *NHEJ1*-related immunodeficiency and its difficult diagnosis due to lack of knowledge.

## Case description

**Figure 1:**
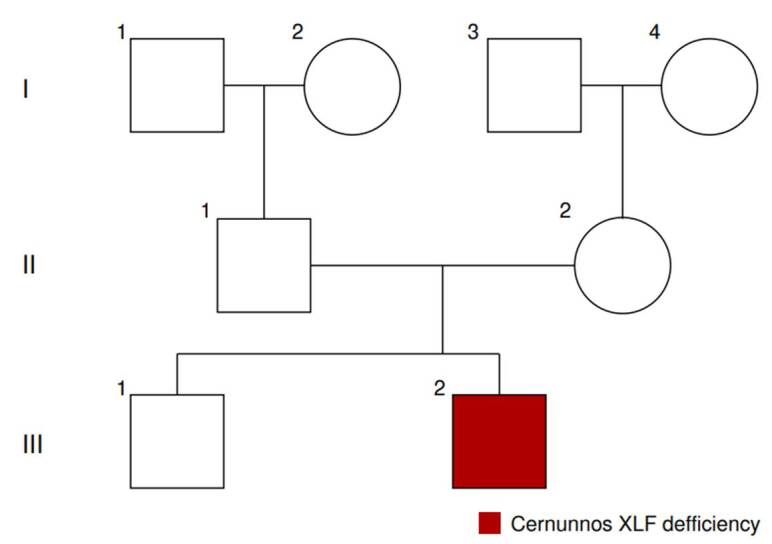
Family tree - Pedigree. III2: 20-year-old male patient, the younger of two brothers, with Cernunnos immunodeficiency due to an homozygous pathogenic variant in *NHEJ1* gene (c.169C>T; p.Arg57Ter). This variant has not been studied in family members. III1: healthy brother, II1 and II2: healthy parents, I1, I2, I3, and I4: healthy grandparents.

**Figure 2:**
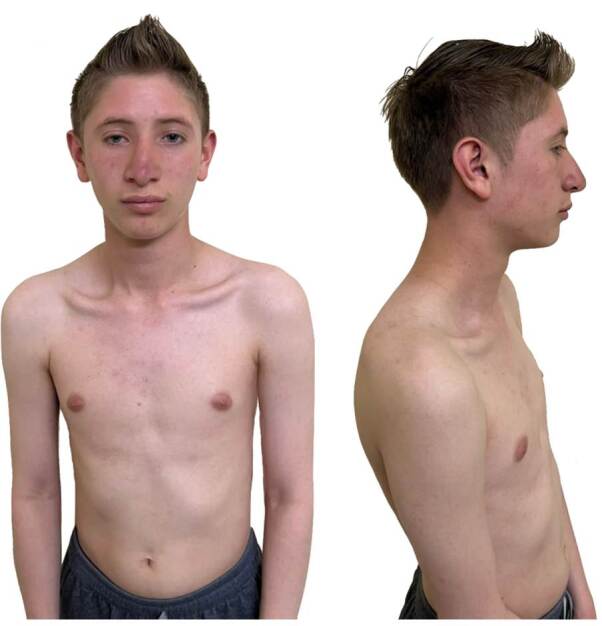
Clinical features pictures. Cernunnos immunodeficiency phenotype in a 20-years-old male patient with a slender body, triangle-shaped face, bulbous nasal tip, thin lips, comedonal lesions on the face, thorax, and back.

We present the case of a twenty-years-old man born to non-consanguineous parents but from the same geographical region: Saboyá (Boyacá) (figure 1) with microcephaly (cephalic perimeter: 49 cm), short stature (153 cm), low weight (39.2 kg) and body mass index of 16.7 kg/m^2^, and a triangle-shaped face (figure 2).

The patient was born by a caesarean section at 36 weeks because of oligoamnios and intrauterine growth retardation. However, he reached his neurodevelopmental goals properly. He has suffered from allergies and bronchitis since he was six months old.

During his childhood, he was hospitalized multiple times due to recurrent sinopulmonary infections, acute otitis media, skin abscesses by *Streptococcus pyogenes*, and pneumonia, sometimes requiring transfusion with red blood cells and platelets. Laboratory results showed lymphocytopenia, neutropenia, and thrombocytopenia. Specific lymphocyte counts and immunoglobulin quantification were performed (table 1) but initially were not conclusive. However, the symptoms of recurrent infections associated with allergies and persistent thrombocytopenia led to suspect a primary immunodeficiency. Therefore, he was treated with 17.5 g of intravenous human immunoglobulin in an eight-hour infusion every 21 days.

**Table 1:** T and B lymphocyte count and immunoglobulin series. Immunoglobulin levels displayed in the laboratory results from 2004 to 2023; T and B lymphocyte count determined in the laboratory results from 2003 to 2023. We observed a substantial reduction on CD4+, CD8+, and CD3+ subpopulations, and total absence of B lymphocytes (CD19+/CD20+).

Ig	Result (mg/dl)	Cells	Recount (cel/μl)	Reference (cel/μl)	Age
IgA	Undetectable	CD3+ (T cells)	1,044	959-2,577	1 year old
IgG	Undetectable	CD4+ (T cells)	726	410-1,590	
		CD8+ (T cells)	270	190-2,300	
IgA	< 60.5	-	-	-	2 years old
IgG	473	-	-	-	
IgM	589	-	-	-	
IgA	40	CD3+ (T cells)	1,063	1,000-2,300	3 years old
IgG	1,266*	CD4+ (T cells)	300	300-1,300	
IgM	327	CD8+ (T cells)	376	150-1,000	
-	-	CD19+/CD20+ (B cells)	22	200-2,100	5 years old
-	-	CD3+ (T cells)	520	800-3,500	16 years old
-	-	CD4+ (T cells)	183	400-2,100	
-	-	CD8+ (T cells)	182	200-1,200	
-	-	CD16+/CD56+ (NK cells)	90.90	70-1,200	
IgA	< 33	CD3+ (T cells)	565	856-2,237	20 years old
IgG	627*	CD4+ (T cells)	195	518-1,472	
IgM	0.9	CD8+ (T cells)	286	205-924	
		CD19+/CD20+ (B cells)	0	100-500	

Ig: Immunoglobin

* Human gammaglobulin infusion

When he became a teenager, he presented a substantial reduction of T lymphocytes (CD3^+^, CD4^+^, and CD8^+^) and an absence of B lymphocytes (CD19^+^/CD20^+^), with normal values of NK cells (CD16^+^/CD56^+^). The management with immunoglobulins was adjusted according to his weight; the latest immunoglobulin regimen was 900 mg/kg of subcutaneous hyaluronidase every 21 days.

The patient’s whole exome was assessed with a next-generation massive sequencer. A total of 6,736 genes were analyzed, including several from the mitochondrial genome, with an average coverage higher than 98% and a minimum depth of 20X.

The analysis identified a nonsense and homozygous mutation in the transcript variant 1 of the *NHEJ1* gene (NM_024782.3), represented by a change in the position 169 of the coding sequence (c.169C>T; p.Arg57Ter; SNP ID: rs118204451). This variant produces a premature stop codon at exon 2, affecting protein functional domains and resulting in a non-functional truncated protein with a decreased ability to repair double-strand DNA breaks. It has a conflicting classification of pathogenicity in the ClinVar database that displays genetic variations and their associated clinical significances in human health.

In the last evaluation in March 2022, two reports gave it the likely pathogenic/pathogenic status, and one reported it as uncertain significance. VarSome (the human genomic variant search engine) classified this variant as pathogenic due to PVS1, PS3, and PM2 criteria according to the variant classification Guidelines of the American College of Medical Genetics and Genomics; the cloud-based Franklin (by Genoox^©^) platform, designed for analyzing and interpreting genetic data, suggests a classification as probably pathogenic with PVS1, and PM2 ACMG criteria.

Hence, at 20 years old, the patient was diagnosed with Cernunnos/XLF deficiency (OMIM: 611290; ClinVar ID: 1323363), included in the Human Gene Mutation Database, and received genetic counseling. Nowadays, he has been receiving immunomodulation therapy with polyclonal immunoglobulin during the follow-up and completing ten years without presenting infections or requiring hospitalization; he goes to university and participates in social and labor domains.

## Discussion

The Cernunnos/XLF deficiency is a severe combined immunodeficiency. It was described for the first time in 2006 ^1^. It is related to the V(D)J somatic recombination, involving the rearrangement of the variable (V), diversity (D), and joining (J) gene loci to produce T- and B-cell antigen receptors ^7^^-^^9^.

The *NHEJ1* gene is located on chromosome 2q35 and has eight exons coding for the 299 amino acid protein denominated non-homologous end joining factor 1. This factor interacts with the X-ray repair crosscomplementing group 4 (XRCC4). At the N-terminal domain, clusters 1-170 are highly preserved and at the C-terminal domain, the 75 clusters of the amino acids are necessary to stimulate the double-strand DNA breaks binding without using a homologous template ^10^^,^^11^. Adequate reparation is crucial to prevent genetic imbalance. Otherwise, the DNA damage may cause replication errors and loss or rearrangements that can lead to mutations and cell death. In some animal models, *NHEJ1* deficiency has been associated with chromosome aberrations as translocations or telomere fusions, resulting in a high risk of developing cancer ^7^^,^^10^.

There are two DNA reparation pathways: the non-homologous (NHEJ) and the homologous recombination. Defects in the homologous recombination pathway result in diseases such as ataxia-telangiectasia, Seckle’s syndrome, Nijmegen’s breakage syndrome, and Fanconi’s syndrome ^1^; the NHEJ pathway factors are considered genomic guardians that guarantee the integrity of the genetic information through the adequate reparation of the DNA lesions ^7^. The damage of any of these proteins (Artemis, DNA ligase IV, DNA-PKCs, XRCC4, and Cernunnos/XLF) implies altered T and B cell maturation ^1^^,^^8^. As NK cells do not perform V(D)J recombination, their counts will be normal ^1^.

The most common *NHEJ1* mutations reported are missense, nonsense, and some splicing site damage. Deletions, insertions, and duplications represent 10.2% of the reported cases. Despite these reported mutations, a correlation genotype/phenotype for the Cernunnos/XLF deficiency has not been established ^4^. Among the clinical manifestations that have been described in Cernunnos/XLF are growth retardation, microcephaly, recurrent infections, combined immunodeficiency with low B and T lymphocytes and normal NK cells, hypogammaglobulinemia, intellectual disability, and increasing sensitivity of their fibroblasts to the gamma ionizing radiation ^4^. These patients have a significant risk of developing malignant tumors, predominantly lymphomas and leukemias ^10^^,^^12^. Then, an early diagnosis, management, and control of comorbidities and recurrent infections are crucial.

The genetic variant found in our patient has been reported in ten cases around the world, where the most common clinical manifestations are microcephaly and hematological disorders ^4^.

For treatment, the autologous transplant of hematopoietic stem cells may be a therapeutic option, but only a few patients have been successfully transplanted ^10^^,^^13^. Jamee et al. published a cohort of 19 patients who had received autologous transplants of hematopoietic stem cells, of which 17 patients (89.5%) survived ^4^. Immunoglobulins should be administered to prevent infectious diseases as the first cause of early death ^12^. Currently, no prenatal diagnosis has been described. However, preconception genetic counseling is recommended for patients with this personal or family medical history because of the 25% probability of autosomal recessive Mendelian inheritance.

In conclusion, patients with a phenotype suggestive of a likely inborn error of immunity should be early-intervened inter- and trans-disciplinary. Specifically, Cernunnos/XLF deficiency should be considered in patients with microcephaly, growth retardation, recurrent infections, and T and B cell alterations like lymphopenia or hypogammaglobulinemia without variation of the NK cells. More studies are required to establish an orientation for diagnosis and treatment, but the autologous transplant of hematopoietic stem cells may be a therapeutic option; we need more clinical trials and protocols or clinical guidelines for radio-exposition regimens.

## References

[1] Çipe FE, Aydogmus C, Babayigit Hocaoglu A, Kilic M, Kaya GD, Yilmaz Gulec E (2014). Cernunnos/XLF deficiency: A syndromic primary immunodeficiency. Case Rep Pediatr.

[2] Tangye SG, Al-Herz W, Bousfiha A, Cunningham-Rundles C, Franco JL, Holland SM (2022). Human inborn errors of immunity: 2022 Update on the Classification from the International Union of Immunological Societies Expert Committee. Immunol.

[3] Turul T, Tezcan I, Sanal O. (2011). Cernunnos deficiency: A case report. J Investig Allergol Clin Immunol.

[4] Jamee M, Khakbazan Fard N, Fallah S, Golchehre Z, Fallahi M, Shamsian BS (2022). Cernunnos defect in an Iranian patient with T- B+ NK+ severe combined immunodeficiency: A case report and review of the literature. Mol Genet Genomic Med.

[5] Revy P, Malivert L, De. Villartay JP (2006). Cernunnos-XLF, a recently identified non-homologous end-joining factor required for the development of the immune system. Curr Opin Allergy Clin Immunol.

[6] Dvorak CC, Cowan MJ. (2010). Radiosensitive severe combined immunodeficiency disease. Immunol Allergy Clin North Am.

[7] Buck D, Malivert LD, Regina B, Fondaneche A, Sanal MC, Ozden PA (2006). Cernunnos a novel non-homologous end-joining factor, is mutated in human immunodeficiency with microcephaly. Cell.

[8] Fayez EA, Qazvini FF, Mahmoudi SM, Khoei S, Vesaltalab M, Teimourian S. (2020). Diagnosis of radiosensitive severe combined immunodeficiency disease (RS-SCID) by comet assay, management of bone marrow transplantation. Immunobiology.

[9] Roch B, Abramowski V, Chaumeil J, Devillartay JP. (2019). Cernunnos/XLF deficiency results in suboptimal V(D)J recombination and impaired lymphoid development in mice. Front Immunol.

[10] Slatter MA, Gennery AR. (2020). Update on DNA-double strand break repair defects in combined primary immunodeficiency. Curr Allergy Asthma Rep.

[11] Dutrannoy V, Demuth I, Baumann U, Schindler D, Konrat K, Neitzel H (2010). Clinical variability and novel mutations in the NHEJ1 gene in patients with a Nijmegen breakage syndrome-like phenotype. Hum Mutat.

[12] Recio MJ, Domínguez-Pinilla N, Perrig MS, Vigil-Iturrate CR, Salmón-Rodríguez N, Faci CM (2019). Extreme phenotypes with identical mutations: Two patients with same nonsense NHEJ1 homozygous mutation. Front Immunol.

[13] Çagdaç D, Õzgür TT, Asal GT, Revy P, De Villartay JP, van Der Burg M (2012). Two SCID cases with Cernunnos-XLF deficiency successfully treated by hematopoietic stem cell transplantation. Pediatr Transplant.

